# Enhanced intratumoral expression of RNF2 is a favorable prognostic factor for patients with cutaneous melanoma?

**DOI:** 10.18632/oncotarget.24825

**Published:** 2018-04-03

**Authors:** Łukasz Kuźbicki, Dariusz Lange, Agata Stanek-Widera, Agnieszka Glińska, Barbara W. Chwirot

**Affiliations:** ^1^ Department of Medical Biology, Faculty of Biology and Environment Protection, Nicolaus Copernicus University, Toruń, Poland; ^2^ Department of Tumor Pathology, Oncology Center – Maria Skłodowska-Curie Institute, Gliwice, Poland

**Keywords:** RNF2 (Ring1B), intratumoral expression, immunohistochemistry, prognostic marker, cutaneous melanoma

## Abstract

Recent studies involving melanoma cell lines suggest that enhanced expression of epigenetic regulator RNF2 supports proliferation and promotes metastasis. However, it is not clear to what extent those data apply to disease progression and prognosis for melanoma patients. Therefore the aim of the present study was to assess the prognostic power of RNF2 intratumoral expression by melanoma cells.

RNF2 was detected immunohistochemically in standard formalin-fixed paraffin-embedded samples of 9 benign nevi, 60 melanomas and 24 nodal metastases.

The lowest percentage of RNF2-positive melanocytes found in nevi was comparable to expression levels in normal skin. The RNF2 expression found in melanomas was significantly higher and it was even more enhanced in metastases. The increased occurrence of RNF2 expressing cells was positively correlated with longer patients’ overall survival. Moreover, a negative correlation was found between intratumoral RNF2 expression and number of generated metastatic lesions.

Our data indicate that development of melanoma is associated with significant changes in RNF2 intratumoral expression and imply that at least for some patients the enhancement of the expression levels of RNF2 in both primary and metastatic lesions may be considered a favorable prognostic factor in melanoma.

## INTRODUCTION

Melanoma causes 90% of skin cancer deaths [1]. Mean survival time of patients with distant melanoma metastases is of the order of 1 year. For that group of the patients the probability of 1 year survival is 45% and the 5 year survival rate is 10% [2]. Despite recent progress in developing new therapies the melanoma survival rates have not changed significantly.

Epigenetic regulation has emerged as an important area in the field of cancer biology with a special focus on a role of epigenetic regulators in controlling mechanisms of cancerogenesis, cancer diagnostics and prognostication. Because of the potential reversibility the epigenetic changes studies of the epigenetic factors may also lead to new forms of cancer therapies [3–5].

RNF2 (Ring1B) is a key element of the Polycomb Repressive Complex 1 (PRC1) but may function as element of other protein complexes. RNF2 has activity of ubiquitin ligase for histone H2A at lysine 119 (H2AK119ub). It is thought that such a monoubiquitination of histone H2A facilitates a contact of histone H1 with nucleosomes as well as reorganization of protein complexes resulting in conformation changes of RNA II polymerase and gene repression [6]. Moreover, RNF2 may also mediate monoubiquitination of the Ambra 1 protein leading to inhibition of a process of autophagy [7]. In some cell types RNF2 may contribute to a degradation of p53 protein by its ubiquitination and/or by stabilization of the Mdm2 protein [8, 9].

Spectrum of potential targets of RNF2 is very wide. Molecular analyses involving melanoma cell lines with enforced RNF2 expression demonstrated a presence of the RNF2 occupancy sites in vicinity of transcription start sites of 3465 genes. Altered expression was detected for 363 of those genes (47% - down-, 53% up-regulated) [10]. It was found that induction of enhanced RNF2 expression supported invasiveness of melanoma cells *in vitro* and metastasis of melanomas in immunodeficient mice. Further research demonstrated that high levels of RNF2 caused strong repression of *LTBP2* gene, a member of latent TGFβ binding family regulating availability of TGFβ ligand. According to the authors [10] the effect of the LTBP2 down-regulation was stimulation of the TGFβ signaling pathway which in turn promoted invasiveness of melanoma cells. The same study demonstrated also that RNF2 supports anchorage independent proliferation of melanoma cells through up-regulation of cyclin D2 due to activation of *CCND2* gene [11]. Another noteworthy conclusion of Rai *et al.* [10] was that the spectrum of genes influenced by RNF2 depends on pre-existing chromatin promoter states of the genes.

The main aim of this work was the assessment of a suitability of intratumoral RNF2 expression in human melanoma as potential prognostic marker. The percentage fractions of the RNF2 – positive cells were determined for both primary and metastatic lesions and correlated with patients’ survival times and with the major clinico-pathological prognostic factors for melanoma.

## RESULTS

**Figure 1:**
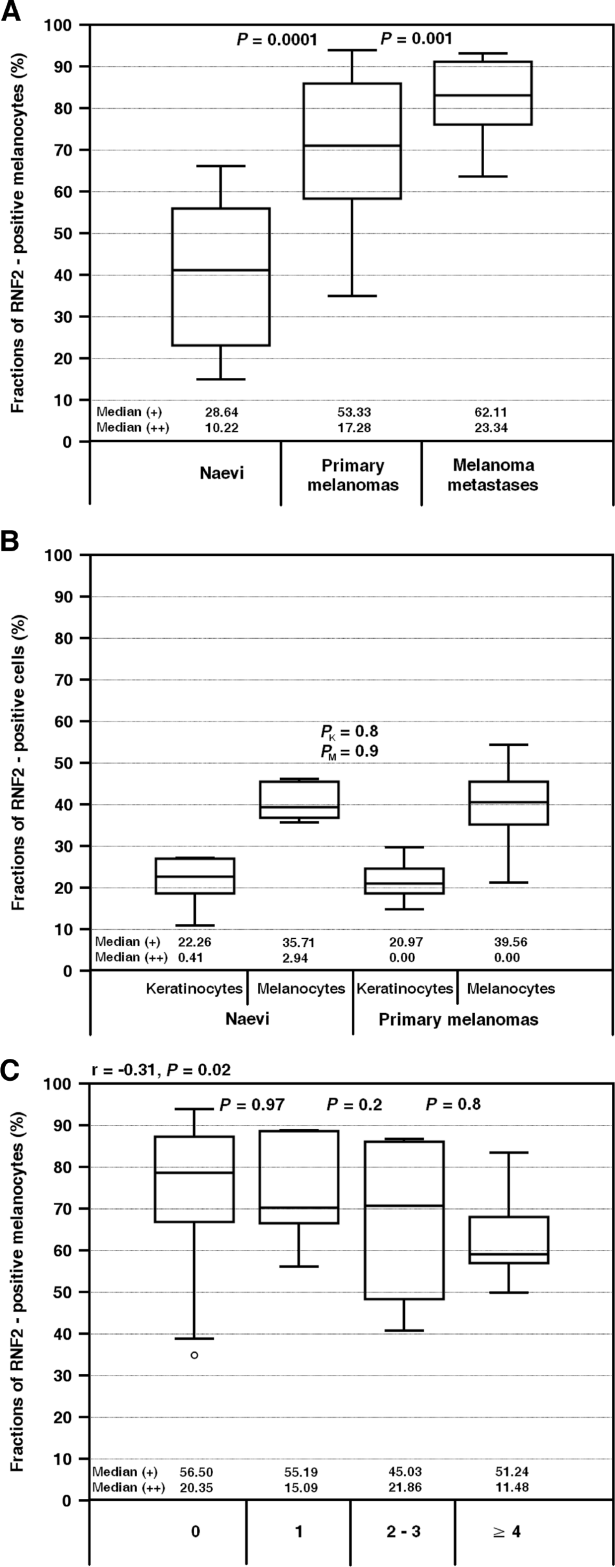
RNF2 expression evaluated on a base of the percentage fractions of the cells with positive staining in: benign nevi, primary melanomas, melanoma lymph node metastases (**A**), normal skin adjacent to nevi and melanomas (**B**) and primary melanomas with different number of lymph node metastases generated by the primary lesions (**C**). Median values (+) and (++) refer to the fractions of the cells with low (+) and high (++) concentration of the reaction product. *P*_K_ and *P*_M_ concern the data obtained for keratinocytes and melanocytes of the normal skin, respectively.

Expression of the RNF2 protein was found in all the lesions investigated. Subcellular localization of immunocomplexes was either nuclear or both nuclear and cytoplasmic. The lowest RNF2 expression was observed in nevi, significantly higher in primary melanomas (even at early stages like I and II Clark level; *P* = 0.006) and the highest in nodal melanoma metastases. The increase of the percentage fractions of the RNF2 – positive cells was paralleled by increase of a contribution of the strongly (++) labeled cells (Figure 1A).

The expression of RNF2 in a normal skin surrounding benign nevi and primary melanomas was detected in ca. 20% of keratinocytes and ca. 40% of melanocytes, usually displaying weak (+) staining intensity (Figure 1B). The highest percentage of RNF2 – positive keratinocytes was observed in the basal layer, lower in the spinous layer and no staining was detected in the cells of the granular layer.

**Figure 2:**
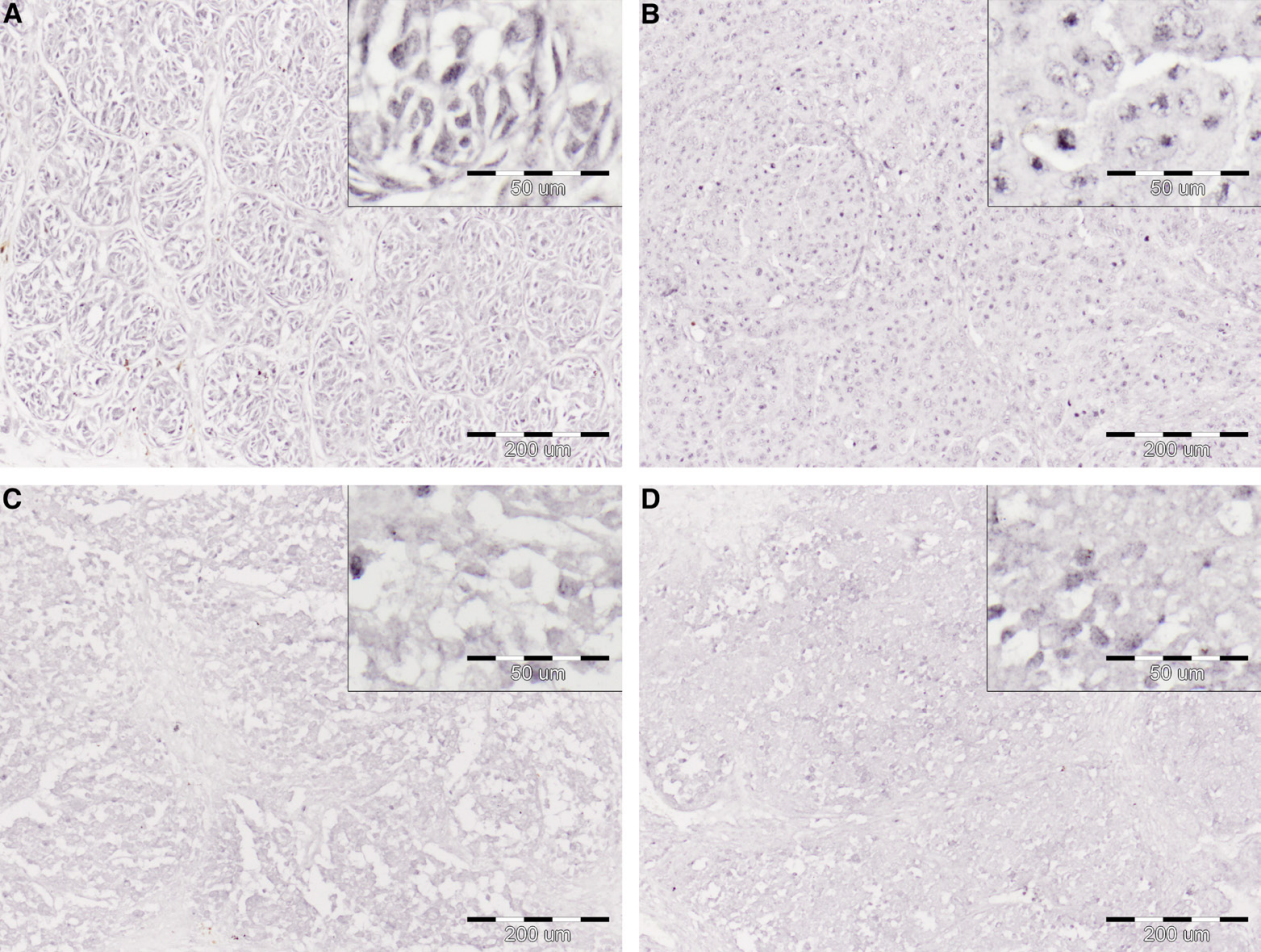
Immunoreactivity for RNF2 in paired nodular primary melanomas (**A, C**) and lymph node metastases (**B, D**). The lesions were obtained from two patients with following melanoma stage and patient’s survival: (1) T1aN1bM1c, 92 months (A, B) and (2) T4aN3M1c, 9 months (C, D). Central regions of the lesions, original magnification 100×, insets 400×.

**Figure 3:**
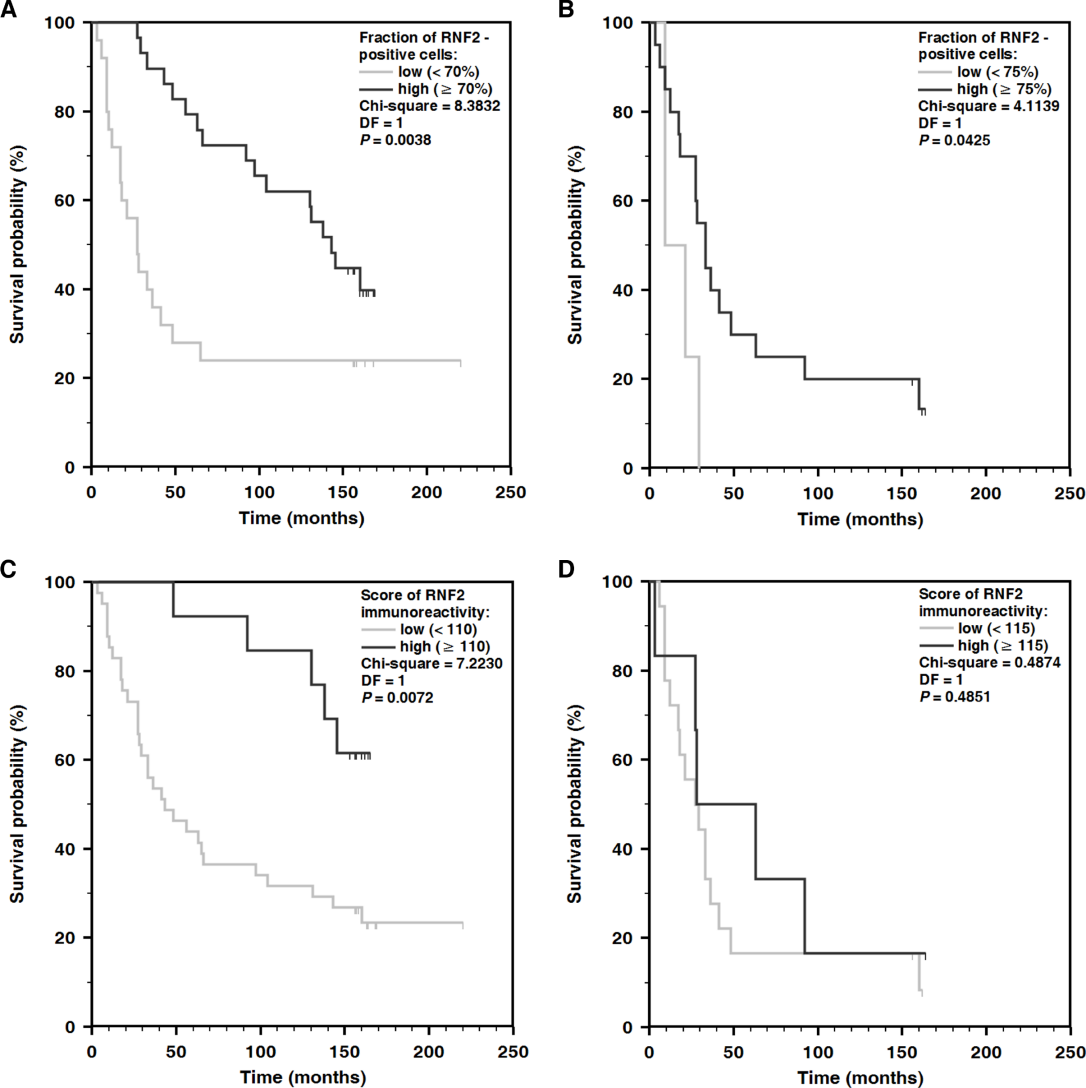
Kaplan–Meier analysis of overall survival and the expression of RNF2 in primary melanomas (**A, C**) and melanoma metastases (**B, D**). The low or high levels of RNF2 expression were evaluated on a base of the percentage fractions of the cells with positive staining (A, B) and of the I parameter accounting also for the staining intensity (C, D).

It may be noteworthy that if the correlated pairs of primary and metastatic melanomas obtained from the same patients are taken into account, the intratumoral RNF2 expression found in the primary lesions tends to decrease slightly with a number of nodal metastases they generated (N0 to N3 according to TNM classification) (Figure 1C). Changes in the expression of RNF2 were independent of the prognostic factors like ulceration (*P* = 0.3), mitotic index (*P* = 0.6), histologic type (*P* = 0.8) and growth phase (*P* = 0.4) of the primary melanomas (Mann–Whitney *U* test). The intratumoral expression of RNF2 was, however, significantly associated with overall survival of the patients. It was found that the survival time drops markedly if the intratumoral expression of RNF2 is less than 70%. At a cut-off threshold of 70% applied to the primary melanomas the mean survival times of the two groups of the patients (*n* = 25 and *n* = 29) differed significantly and were equal to 58 and 119 months, respectively (Figures 2, 3A Table 1). For metastatic lesions the cut-off threshold of 75% was needed to find significant differences in prognoses. The mean survival time found for 4 cases with the expression below the threshold was 17 months while for 20 cases with at least 75% of the cells expressing RNF2 it was equal to 57 months (Figures 2, 3B, Table 1). It seems interesting that the prognostic value of the RNF2 expression was dependent not so much on the expression level found for individual cells but rather on the intratumoral expression by high fractions of the tumor cells. If the parameter I accounting for both the intratumoral expression and for contributions of the cells with the strong (++) staining was used the correlation with the patients’ survival was found only for the primary lesions (Figure 3C, 3D, Table 2). Cox proportional hazard analysis was used to determine the value of the RNF2 expression as a prognostic marker independent of other parameters including sex, age (18–45, 46–54 and more than 55 years) [12] as well as of a postoperative treatment with chemo-, radio- or immunotherapy. Such modelling demonstrated that the correlation of lower expression levels of RNF2 and worse prognoses is independent of the parameters investigated as evidenced by the low values of the *P* parameter (Table 3).

**Table 1:** Percentage fractions of the RNF2 – positive cells in melanomas and patients’ overall survival

Type of lesion	Primary melanomas		Melanoma metastases	
Percentage fraction of the stained cells	Low (<70%) *n* = 25	High (≥70%) *n* = 29	Low (<75%) *n* = 4	High (≥75%) *n* = 20
Mean survival time (months)	58	119	17	57
Median survival time (months)	27	143	15	33
Hazard ratio	2.4977		2.7801	
95% Confidence interval	1.4107–5.9660		1.0604–30.7439	

**Table 2:** Score of RNF2 immunoreactivity (I value) in melanomas and patients’ overall survival

Type of lesion	Primary melanomas		Melanoma metastases	
Value of I parameter	Low (<110) *n* = 41	High (≥110) *n* = 13	Low (<115) *n* = 18	High (≥115) *n* = 6
Mean survival time (months)	75	140	46	63
Median survival time (months)	43	156	28	45
Hazard ratio	3.3216		1.4144	
95% Confidence interval	1.2952–5.2201		0.5416–3.6388	

**Table 3:** Statistical significance of correlations between patients’ overall survival and expression of RNF2 in primary melanomas including selected characteristics of patients using Cox proportional hazards modeling

Intratumoral expression of RNF2	Characteristics of patients		
	Sex	Age	Therapy
Percentage fraction of the stained cells	*P* = 0.0015	*P* = 0.0005	*P* = 0.0028
Score of immunoreactivity	*P* = 0.0018	*P* = 0.0034	*P* = 0.0020

**Figure 4:**
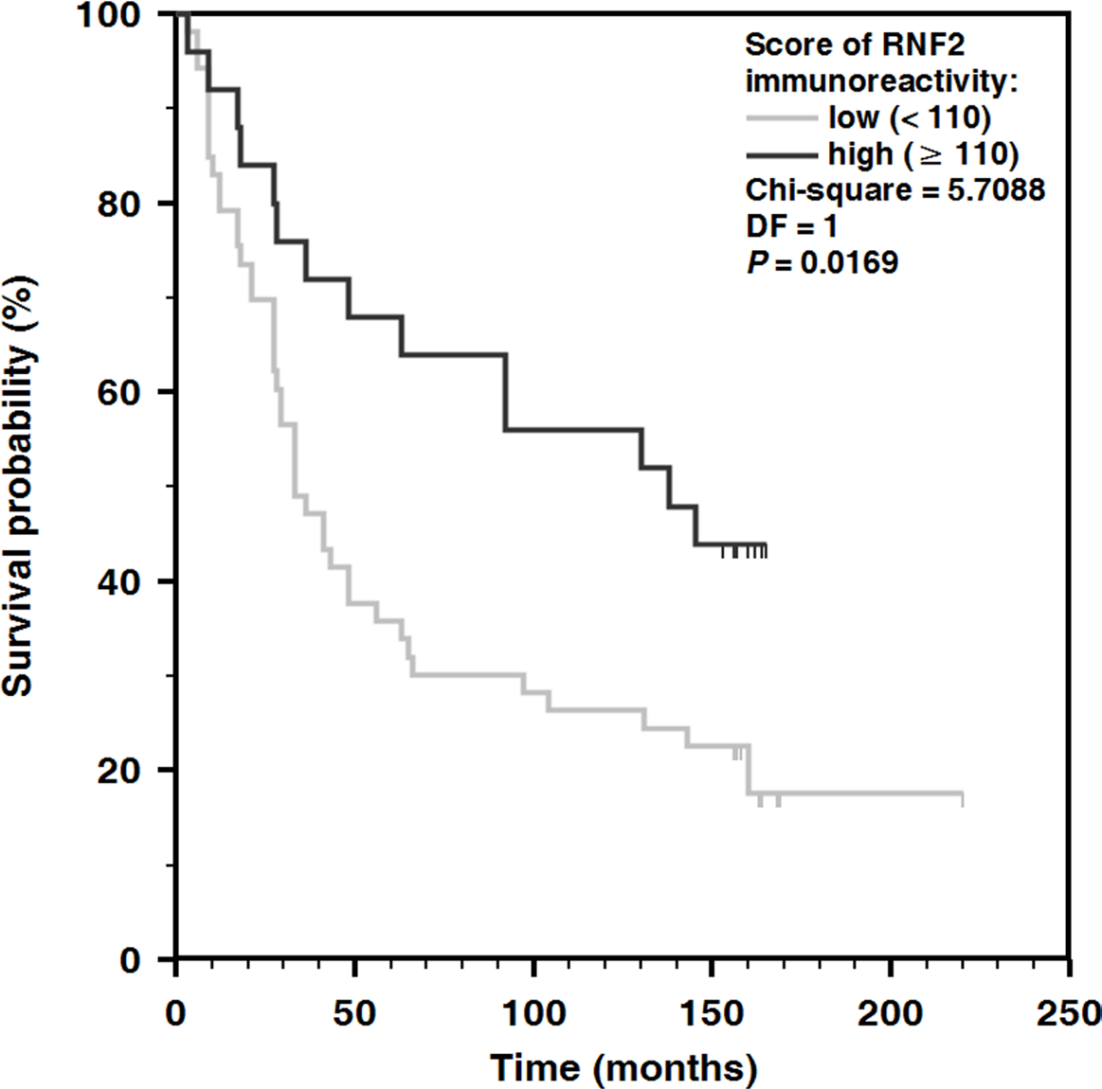
Kaplan–Meier analysis of overall survival and the expression of RNF2 in a group comprising both primary melanomas and metastases The low or high levels of RNF2 expression were evaluated on a base of the I parameter accounting for both the percentage fractions of the cells with positive staining and the staining intensity.

For a sake of comparison between our results on the prognostic value of the RNF2 expression in melanoma and the data published earlier [10], we analyzed also the correlation between the I parameter values determined for the primary and metastatic lesions taken as one group and the patients’ survival. Contrary to the conclusions of Rai *et al.* [10] we found that the cases with low expression of RNF2 have a worse prognosis (Figure 4).

## DISCUSSION

We show in this work that expression of RNF2 by melanocytes of benign nevi and of a normal skin is similar. In both cases the expression was observed on average for 40% of the cells while for keratinocytes the mean percentage fraction of the RNF2 – positive cells was ca. 20% and was highest in the basal layer. Similar observation for normal human skin was reported by Sánchez-Beato *et al.* [13] who carried out immunohistochemical studies of RNF2 expression in tissue microarrays.

The expression of RNF2 in primary melanomas was significantly higher with 70% of the positively stained melanocytes and increased to 80% of RNF2 – positive cells in nodal metastases. Development and progression of melanoma from primary to metastatic lesions were accompanied also by increase of percentage of the cells with a strong (++) staining. Similar observation was described by Rai *et al.* [10] who found the lowest expression of RNF2 in nevi, much stronger in primary melanomas and the highest in melanoma metastases. Such findings might suggest an association between the expression of RNF2 by the tumor cells and the disease progression. However, analysis of the intratumoral expression of RNF2 in a relation to the patients’ survival demonstrated that high expression levels exceeding 70% of cells of primary melanomas and 75% of cells of the nodal metastases were associated with a longer survival. Such a beneficial effect of high RNF2 expression found for both the primary and metastatic melanomas is much stronger in the case of primary lesions but is clearly observed also if one analyses both the types of the lesions as one group. The latter approach might seem to be controversial, however, since it was applied by Rai *et al.* [10] we had to use it too to compare the results of the two studies.

Our data suggest that a determination of percentage fractions of the RNF2 – positive cells seems to be the optimum algorithm for evaluation of the intratumoral expression of the protein. Using the scoring system accounting for the staining intensity of individual cells did not result in a better determination of a role of RNF2 levels as melanoma prognostic factor.

We also found that the intratumoral expression of RNF2 decreases slightly in the primary tumors with increasing numbers of nodal metastases produced by those lesions.

Interestingly several studies published up to now described worsening of prognoses with increased RNF2 expression in cancers of pancreas [14], esophagus [15], liver [16], urinary bladder [17] and ovary [18]. It is possible that spectrum of genes actively modulated by RNF2 changes in different cell types [10, 19, 20].

Taken together our results support observations of Rai *et al.* [10] of enhancement of the RNF2 expression associated with a malignant transformation of melanocytes. Higher expression levels of the protein are reflected in significant increase in the percentage of the RNF2 – positive cells, also those with a strong staining, already in the early stage melanomas compared to nevi and normal skin. Similar to Rai *et al.* [10] we also found further increase of the RNF2 expression at transition from primary to metastatic lesions.

The most significant result of the present study seems to be, however, finding that enhanced level of the intratumoral RNF2 expression may be a favorable prognostic indicator for the patients with both primary melanomas and nodal metastases. Our conclusion contradicts that of Rai *et al.* [10] who found that at *in vitro* conditions the induced enhanced expression of RNF2 supported both proliferation and invasiveness of cultured melanoma cells. The same authors also presented Kaplan-Meier curves generated using the TCGA survival data for the three groups of the patients defined by amplification/deletion of copy numbers and RNF2 expression changes [10]. According to those data both the amplification and upregulation of RNF2 expression (12 and 18 cases, respectively) as well as the deletion and downregulation (2 and 4 cases) of the expression levels were associated with a shorter survival compared to a normal group with no copy number/expression changes (44/104 cases). It should be emphasized, however, that the TCGA data were obtained using molecular biology techniques. Therefore, the survival analysis of Rai *et al.* [10] cannot be directly compared with our results based on immunohistochemical analysis of the intratumoral RNF2 expression assessed by a determination of the percentage fractions of the RNF2 – positive cells.

Our work demonstrates that at least in some patients the increased intratumoral expression of RNF2 seems to improve the prognoses. As pointed out by Rai *et al.* [10] the spectra of genes regulated by RNF2 are strongly dependent on pre-existing chromatin promoter states. It is possible that it is because of such a dependence that our data disaccord with the results of Rai *et al.* [10] with respect to prognostic meaning of the increased RNF2 expression in melanoma.

## MATERIALS AND METHODS

### Patient material

The material for study was formalin-fixed paraffin-embedded tissue samples obtained from archives of Department of Tumor Pathology, Oncology Center – Maria Skłodowska-Curie Institute, Gliwice, Poland. The research has been approved by Bioethics Committee at the Oncology Center (Ref. No. KB/430/27/14). The study consisted of 60 primary melanomas, 24 lymph node metastases correlated with their primary counterparts and 9 benign nevi (junctional, 1; compound, 3, blue, 1; Spitz, 1 and dysplastic, 3). Primary melanomas were classified according to Clark infiltration levels (I, *n =* 1; II, *n =* 12; III, *n =* 28; IV, *n =* 11; V, *n =* 8), Breslow thickness (≤1.0 mm, *n =* 15; >1.0 – 2.0 mm, *n =* 11; >2.0 – 4.0 mm, *n =* 15; >4.0 mm, *n =* 19), ulceration (absent, *n =* 21; present, *n =* 34), mitotic index (≤1 mitosis/mm^2^, *n =* 12; >1 mitosis/mm^2^, *n =* 42), histologic type (superficial spreading, *n =* 28; nodular, *n =* 30; acral, *n =* 1), growth phase (RGP, *n =* 26; VGP, *n =* 28) and a number of lymph nodes with metastases assigned to individual primary lesions (0, *n =* 28; 1, *n =* 9; 2–3, *n =* 4; ≥4, *n =* 11). All the lesions investigated were classified histologically. Complete clinical data including survival times were available for 54 of 60 melanoma patients. The age of the patients varied in a range of 21–82 years (mean – 54; median – 53 years). None of the patients was subject to any form of therapy before the resection of the tumors. Thereafter some of the patients were treated with chemotherapy (*n =* 11), radiotherapy (*n =* 16) or immunotherapy (*n =* 5).

### Immunohistochemistry

The 3 μm sections were mounted on 3-aminopropyltriethoxysilane (APTES; Sigma-Aldrich, St. Louis, Missouri, USA) coated slides. Tissue sections were deparaffinized, rehydrated, treated with 0.01M citrate buffer, pH 6.0 at 90° C for 60 min. and incubated with 0.3% hydrogen peroxide (Sigma-Aldrich) at room temperature for 30 min. Non-specific binding of antibodies was blocked by incubating the sections with 1.5% normal goat serum (Vector Laboratories, Burlingame, California, USA) for 30 min. The tissue sections were then treated with primary polyclonal rabbit anti-RNF2 antibodies (HPA026803, Sigma-Aldrich) in PBS, pH 7.4 (1:100 v/v) with 0.1% (w/v) BSA (Vector Laboratories) and incubated for 60 min. at room temperature in humid chamber. Immunoreacitve complexes were detected using secondary goat anti-rabbit antibodies labeled with biotin and ABC complex with horseradish peroxidase (Vectastain Elite ABC Kit, Vector Laboratories) according to manufacturer’s protocol. The substrate for peroxidase was 0.05% 3.3’-diaminobenzidine (DAB; Sigma-Aldrich) with 0.01% hydrogen peroxide (Sigma-Aldrich) and 0.06% nickel chloride (II) (Sigma-Aldrich). Negative control sections treated as in normal protocol but with PBS pH 7.4 with 0.1% (w/v) BSA (Vector Laboratories) instead of primary antibodies solution did not produce detectable staining. Staining of epidermal keratinocytes and melanocytes of normal skin surrounding the lesions constituted an intrinsic positive control.

### Immunohistochemical scoring

Microscopic examinations were carried out using a BX61 microscope (Olympus Corporation, Tokyo, Japan). Images were recorded with an XC50 camera (Olympus Soft Imaging Solutions, Münster, Germany) and imagine Cell^P^ 3.3 software (Olympus Soft Imaging Solutions, Hamburg, Germany, 2009).

Semi-quantitative analysis of immunohistochemical staining involved counting of cells in three fields of view, in central regions of the lesions, separately for each of the skin layers, at 1000× magnification. The staining intensity was evaluated too and classified as weak (+, blue) or strong (++, dark blue/black).

Mean percentage fractions of all the RNF2 – positive cells were calculated as a measure of intratumoral expression of the protein. Additionally, to account for different staining intensities the labeling index I [21] was also calculated as a sum of the intensities of staining (1 or 2) multiplied by the percentage of positive cells, respectively (+) and (++).

### Statistical analysis

The data on RNF2 expression are presented as box plots with marked median values of percentage fractions of the stained cells. Statistical significance of the results was evaluated using Mann–Whitney *U* test with *P* value of less than 0.05. The correlation between the RNF2 expression in primary melanomas and number of lymph nodes with metastases generated by the primary lesions was assessed by calculating Spearman correlation coefficient. The probability of overall survival was evaluated for both the percentage fraction of the RNF2 – positive cells and the I parameter values using Kaplan-Meier method. The log-rank test was used for comparisons of the survival curves while Cox proportional hazards model was applied to assess the importance of characteristics of the patients including sex, age and postoperative treatment in the survival times (MedCalc Software, Ostend, Belgium).
